# Ginsenoside F1-Mediated Telomere Preservation Delays Cellular Senescence

**DOI:** 10.3390/ijms241814241

**Published:** 2023-09-19

**Authors:** Jingang Hou, Yeejin Yun, Byeongmin Jeon, Jongin Baek, Sunchang Kim

**Affiliations:** 1 Intelligent Synthetic Biology Center, 291 Daehak-ro, Yuseong-gu, Daejeon 34141, Republic of Korea; houjg2022@guest.kaist.ac.kr (J.H.); jbm0901@kaist.ac.kr (B.J.); baekji@kaist.ac.kr (J.B.); 2 Department of Biological Sciences, Korea Advanced Institute of Science and Technology, 291 Daehak-ro, Yuseong-gu, Daejeon 34141, Republic of Korea; yyj07@kaist.ac.kr

**Keywords:** ginsenoside F1, TRF2, mitochondria, oxidative stress, senescence

## Abstract

Telomeres play pivotal roles in processes closely related to somatic senescence and aging, making them a compelling target for interventions aimed at combating aging and age-related pathologies. Ginsenoside, a natural compound, has emerged as a potential remedy for promoting healthy aging, yet how it protects telomeres remains incompletely understood. Here, we show that treatment of F1 can effectively restore the level of TRF2, thereby preserving telomere integrity. This restoration leads to inhibition of the DNA damage response and improvements in mitochondrial function and, ultimately, delays in cellular senescence. Conversely, depletion of TRF2 causes mitochondrial dysfunction, accompanied by increased oxidative stress, autophagy inhibition, insufficient energy metabolism, and the onset of cellular senescence. These observations underscore the critical role of TRF2 in maintaining telomere integrity and direct association with the initiation of cellular senescence. We conduct a further analysis, suggesting F1 could bind in proximity to the TRF2 heterodimer interface, potentially enhancing dimerization stability. These findings suggest that F1 may be a promising natural remedy for anti-aging, and restoring TRF2 could potentially prevent telomere-dependent diseases commonly associated with the aging process.

## 1. Introduction

Studies on tissues with high cell turnover rates, such as the intestine and testis, support the notion that telomere length is a determinant for tissue fitness in organisms [1,2]. Telomere shortening is thus one of the most frequently cited challenges, which occurs concomitantly with some well-documented diseases such as dyskeratosis congenital, pulmonary fibrosis, aplastic anemia, and cancers [3,4,5,6]. Several hallmarks of aging have been proposed, such as genomic instability, stem cell exhaustion, mitochondrial dysfunction, cellular senescence, and telomere dysfunction [7]. Interestingly, telomeres interrelate with other hallmarks, serving to drive or amplify these mechanisms, and those lower than the critical length of telomere repeats undergo telomere dysfunction and activate DNA damage pathways, leading to replicative senescence or death in both somatic and stem cells [8,9]. Experimental disruption of telomere function causes genomic instability, as evidenced by cytogenetic analysis, which includes amplification, deletions, translocation, and cancer initiation [10]. Accumulated cellular reactive oxygen species (ROS) caused by decreased function in mitochondria give rise to lesions in telomere DNA, driving the premature aging phenotypes [11]. Several findings have highlighted the vital roles of telomeropathy in premature aging syndromes and age-associated diseases. During physiological aging, kidneys undergo negative structural and functional alterations including atrophy, sclerosis, and fibrosis and develop acute kidney injury, glomerulonephritis, and chronic kidney disease, in which telomere dysfunction and cellular senescence have a key contribution [12,13]. Brain aging is the most important risk factor for neurodegenerative disorders. In line with this, the telomere length has been shown to positively correlate with improved memory and cognition in patients with amyloid pathology [14] and recapitulate the Alzheimer’s disease phenotype in a rodent model [15]. Cardiovascular disease is the leading cause of mortality in Western countries. Studies have shown that the telomere length correlates with the severity of hypertrophic cardiomyopathy, and telomere dysfunction has been identified as a potential driver of atherosclerosis [16]. Consistent observations have revealed that alterations in telomeres across various aging tissues lead to genomic changes, functional decline, and ultimately cellular dysfunction and apoptosis. Telomeric repeat-binding factor 2 (TRF2) plays a pivotal role in the formation and maintenance of the ‘t-loop’ structure, safeguarding telomeres from being identified as DNA double-strand breaks. Such misidentification would normally activate DNA damage responses, leading to cellular senescence. Therefore, TRF2 assumes a critical responsibility in preserving the structural and functional integrity of telomeres [17]. During replicative senescence characterized by activated p53, endogenous TRF2 experiences downregulation, and research has elucidated a positive feedback loop existing between p53 and TRF2, which contributes to telomere damage signaling and the onset of cellular senescence [18]. As such, it is conceivable that employing precision-based strategies to modulate TRF2 could potentially yield favorable outcomes in mitigating cellular senescence and further delaying or preventing the progression of aging and age-associated pathologies.

Our previous studies have demonstrated that ginsenoside F1, the metabolite of Rg1, is one of the most important constituents of Panax ginseng. It is a saponin with a low molecular mass (~640 Da) which exhibits neuroprotective properties against aged human astrocytes and the adverse effects of a high-fat diet on the mouse brain. In addition, the findings indicated that the beneficial effects of ginsenoside F1 extended to an improvement in the amyloid precursor protein/presenilin 1 (APP/PS1) transgenic mouse model of Alzheimer’s disease [19,20,21,22], In this study, our objective was to evaluate the potential impact of F1 on telomeres, as well as investigate the subsequent effects on mitochondrial function and alterations in the profile of cellular senescence. We focused on specific human primary somatic cells derived from tissues with relatively lower proliferative rates but higher vulnerability to fragility, namely the brain, kidney, and heart. Our findings offer direct empirical support indicating that F1 exhibits a significant enhancement in telomere maintenance achieved through the preservation of TRF2. This preservation of the telomere structure contributes to the attenuation of the aging process.

## 2. Results

### 2.1. Ginsenoside F1 Preserved the Telomere Length in Aged Somatic Cells

**Figure 1:**
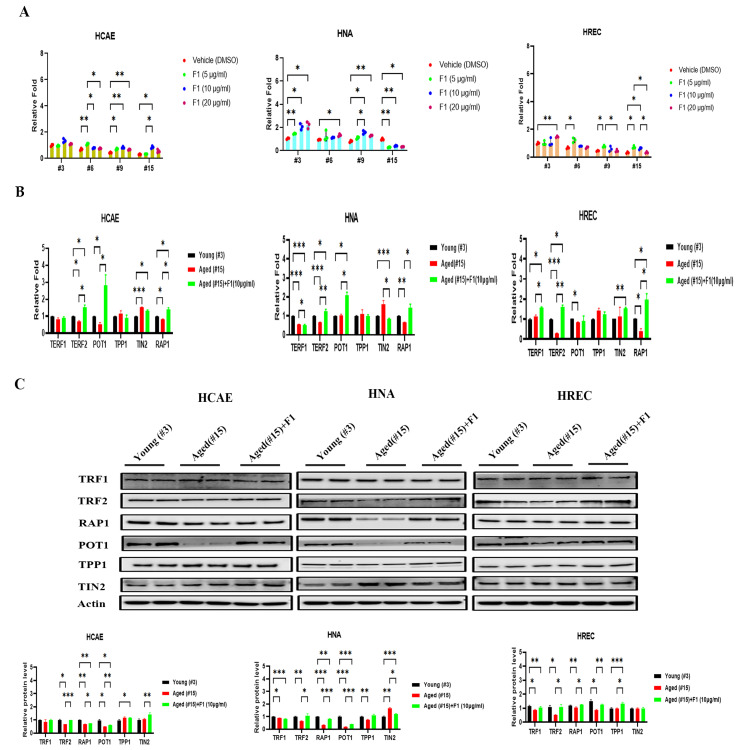
Ginsenoside F1 preserves telomere function in aged somatic cells. (**A**) Telomere length assay of somatic cells of different ages (passages). (**B**) qRT-PCR analysis of shelterin proteins. (**C**) Western blot analysis of shelterin proteins. Results are expressed as mean ± SEM. Statistical significance was determined by one-way ANOVA (* *p* < 0.05, ** *p* < 0.01, and *** *p* < 0.001). We used the Shapiro–Wilk test to test the normality of the distribution. All the data were in accordance with the normal distribution. All the data were compared by one-way analysis of variance followed by a Tukey post hoc test for multiple comparisons.

Serially grown normal somatic cells are a well-characterized model system for mimicking the cellular and molecular changes associated with development and aging. To address the possible impact of ginsenoside F1 on normal aging, we performed assays on the telomere length at population passages of 3, 6, 9, and 15 in three normal primary somatic cell lines, including human astrocytes (HNA), human cardiac coronary artery endothelial cells (HCAE), and human renal proximal tubule epithelial cells (HREC). As expected, the telomere length shortened as passages increased in all three somatic cell lines (Figure 1A). There were significant differences between the ginsenoside F1-treated and vehicle-treated cells, especially at 9 and 15 passages, reaching a maximum effect at a concentration of 10 μg/mL and a slight decrease at 20 μg/mL. We used 10 μg/mL of F1 in the subsequent experiments.

We further analyzed both the transcriptional and translational levels in six major proteins of the shelterin complex: telomeric repeat-binding factor 1 (TERF1), telomeric repeat-binding factor 2 (TERF2), TRF1-interacting nuclear protein-2 (TIN-2), protection of telomeres 1 (POT1), repressor and activator protein 1 (Rap-1), and TPP1 or adrenocortical dysplasia protein homolog (ACD). For the mRNA expression level (Figure 1B), TERF2 and RAP1 were dramatically reduced in the aged cells. However, this was restored in all three cell lines after the F1 challenge. Interestingly, an increase in TERF1 was only observed in HREC, while there was a decrease in HNA. For the protein expression level (Figure 1C), TRF2, RAP1, and POT1 were markedly decreased in the aged cells compared with the early three-passage cells, while these were inhibited by F1 treatment. Thus, these results indicate that F1 regulates telomere function via the shelterin complex in somatic cells, and this appears to be age-dependent.

### 2.2. Ginsenoside F1 Modulated the Protein Kinase B (AKT) and Mitogen-Activated Protein Kinase (MAPK) Pathways in Aged Somatic Cells

**Figure 2:**
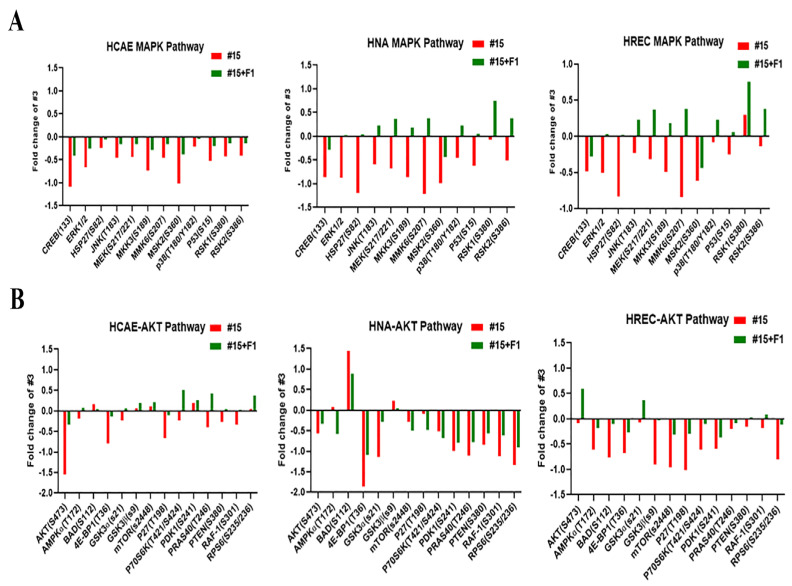
Ginsenoside F1 regulates MAPK and AKT pathways in aged somatic cells. (**A**) Antibody array analysis of MAPK pathways. (**B**) Antibody array analysis of AKT pathways.

To address the role of potential signaling pathways during telomere dysfunction in aged cells, we measured the levels of 12 and 14 phosphorylated proteins using MAPK and AKT antibody arrays, respectively (Figure 2). Of the factors detected by the arrays in HCAE, F1 reduced the suppressed phosphorylation of 12/12 in the MAPK signaling pathways and 9/14 in the AKT signaling pathways. In HNA, F1 elevated the phosphorylation of 10/12 in the MAPK signaling pathway while suppressing the reduction of 8/14 in the AKT signaling pathways. In HREC, F1 enhanced the phosphorylation of 10/12 in the MAPK signaling pathways while repressing the reduction of 14/14 in the AKT signaling pathways. Notably, in all the aged primary cells, several phosphorylated proteins of the MAPK signaling pathways were consistently suppressed, including CREB, ERK, HSP27, MEK, MMK6, MSK2, and RSK2, and as for the AKT signaling pathways, typical factors such as AKT, 4E-BP1, P70S6K, PRAS40, PTEN, and RPS6 were generally blocked. Importantly, most of these proteins are closely associated with cell proliferation and shelterin protein stability, which are profoundly alleviated by F1. Thus, F1 may exert telomere protection through the modulation of AKT and MAPK signaling pathways.

### 2.3. Ginsenoside F1 Ameliorated Mitochondrial Dysfunction and Cellular Senescence in Aged Somatic Cells

**Figure 3:**
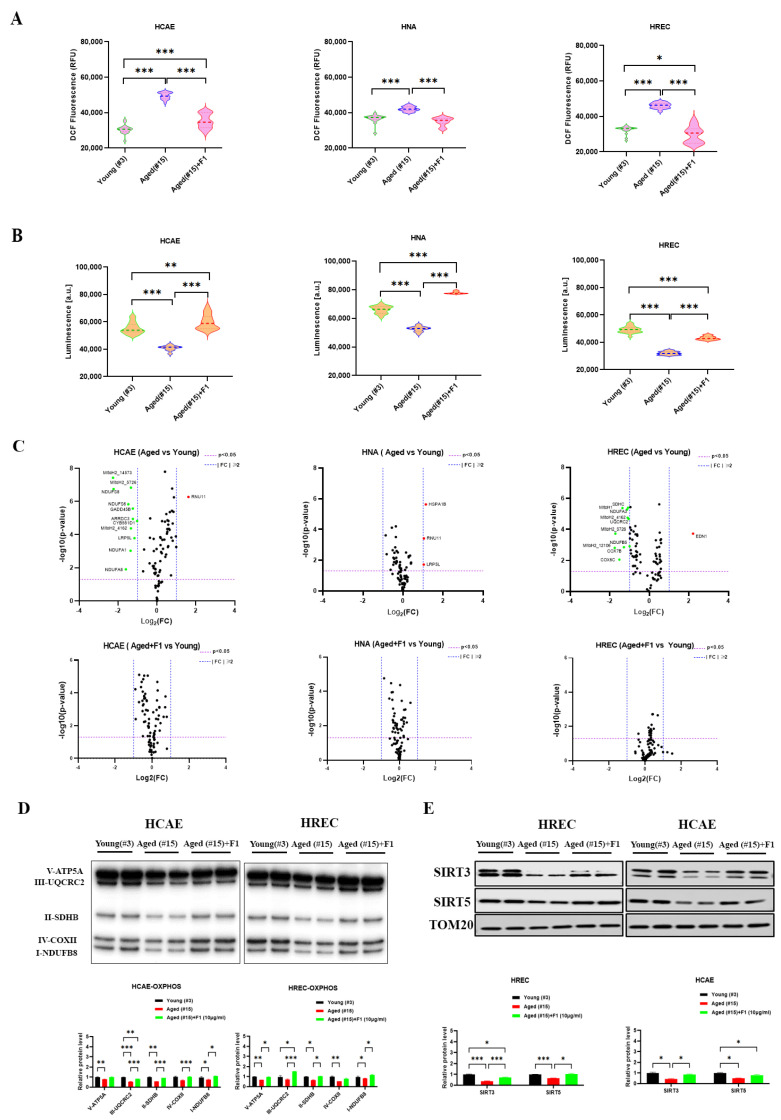
Ginsenoside F1 mitigates mitochondrial dysfunction in somatic cells. (**A**) ROS level was measured using CM-H2DCFDA. (**B**) Quantification of ATP level was measured using luminescent ATP detection assay. (**C**) PCR array of mitochondrial energy metabolism pathways. (**D**) Immunoblots of OXPHOS complexes in mitochondrial preparation. (**E**) Immunoblots of SIRT3 and SIRT5 in mitochondrial preparations. Results are expressed as mean ± SEM. Statistical significance was determined by one-way ANOVA (* *p* < 0.05, ** *p* < 0.01, and *** *p* < 0.001). The Shapiro–Wilk test was used to test the normality of the distribution. All the data are in accordance with the normal distribution. All the data were compared by one-way analysis of variance followed by a Tukey post hoc test for multiple comparisons.

Telomere damage is closely connected with mitochondrial dysfunction, a hallmark of aging. To investigate whether and how F1 treatment impacts mitochondrial dysfunction, we analyzed the ROS and adenosine triphosphate (ATP) levels in the aged somatic cells (Figure 3A,B). As predicted, the aged cells presented elevated ROS and a decreased level of endogenous ATP, which was effectively reversed by F1 exposure.

To decipher how F1 regulated mitochondrial energy metabolism, we determined the levels of 84 genes involved in the pathways (Figure 3C). All the data were normalized by five housekeeping genes. The aged cells showed few changes in HNA, with only a significant increase in HSPA1B, RUN11, and LRP5L, indicating that few dysfunctions occurred in the aged astrocytes. On the contrary, remarkable changes were seen in the aged HCAE and HREC. For the HCAE, genes detected with a statistical decrease included MitoH2_14573, MitoH2_5726, MitoH2_4162, NDUFS8, GADD45B, ARRDC3, CYB56D1, LRP5L, NDUFA1, and NDUFA8, while only RNU11 was significantly increased. For the HREC, the genes downregulated at significant levels in the aged cells were NDUFA3, SDHC, UQCRC2, NDUFB5, COX7B, COX6C, MitoH1, MitoH2_4162, MitoH2_5726, and MitoH2_12106, whilst EDN1 was the only upregulated one. Strikingly, treatment with F1 substantially restored these changes to normal levels (three) in aged cells.

To confirm whether the observed upregulation of genes involved in mitochondrial respiration correspondingly changed protein expression, we performed Western blotting on mitochondrial complexes using an antibody cocktail against mitochondrial oxidative phosphorylation complexes (OXPHOS) (Figure 3D). The aged cells contained reduced levels of subunits of complexes I, II, III, IV, and IV compared with the young cells. However, the aged cells treated with F1 decreased to a lesser extent.

To analyze the changes of potential regulators in the mitochondria of aged cells, we evaluated the protein levels of SIRT3 and SIRT5 in mitochondrial preparations (Figure 3E). In the HREC, reduced levels of SIRT3 and SIRT5 in aged cells were restored by F1 treatment, indicating that F1 is partially implicated in the regulation of enzymes in mitochondrial function improvement.

**Figure 4:**
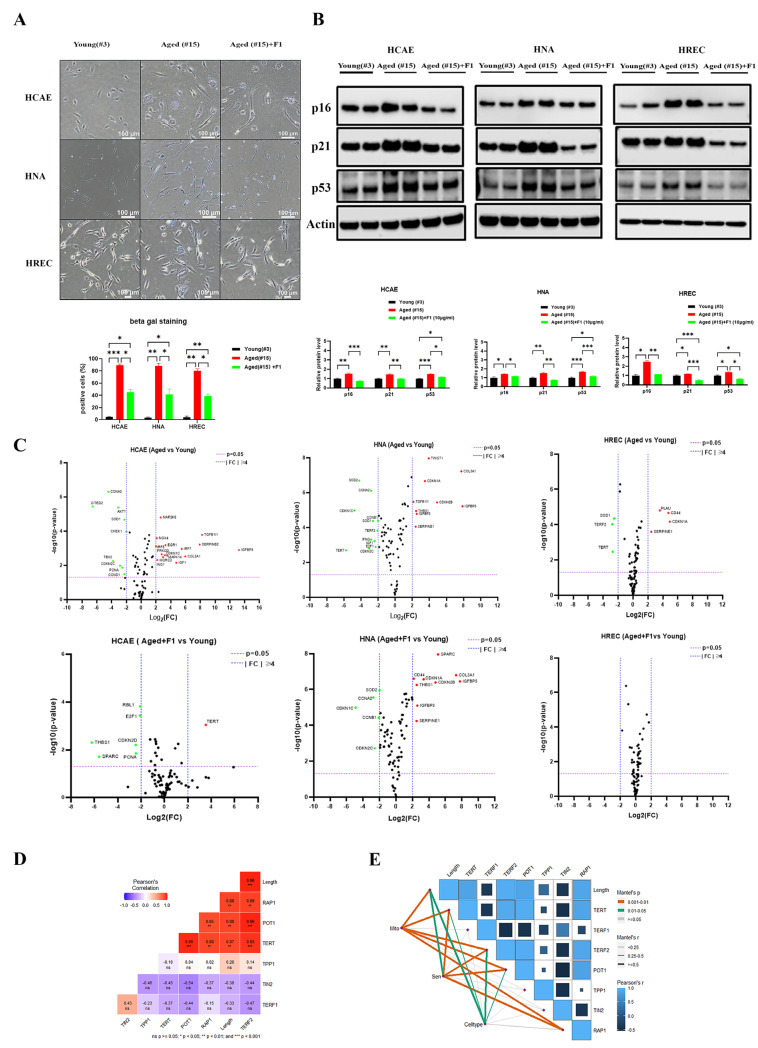
Ginsenoside F1 delays cellular senescence in somatic cells. (**A**) Images of beta gal staining and quantification. (**B**) Immunoblots of senescence markers p16, p21, and p53. (**C**) PCR array of cellular senescence pathways. (**D**) Heatmap presents the matrix of Pearson correlation coefficients among telomere length, TERF1, TERF2, POT1, TIN2, TPP1, and RAP1. Significant differences were indicated by *p* values (ns = not significant, * *p* < 0.05, ** *p* < 0.01, and *** *p* < 0.001). (**E**) Spearman’s correlation analysis and Mantel tests for genes involved in mitochondrial energy metabolism (Mito), cellular senescence (Sen), and somatic cell types (Celltype). The color of the line represents the significance of the differences (*p* values). The size of the line represents correlation coefficients (Mantel’s r). We used the Shapiro–Wilk test to test the normality of the distribution. All the data are in accordance with the normal distribution. All the data were compared by one-way analysis of variance followed by a Tukey post hoc test for multiple comparisons.

Critical short telomeres and ROS-mediated damage to telomeric DNA initiate cellular senescence. We therefore examined the levels of senescence markers using beta galactosidase staining and the Western blot method. A significant increase in the number of beta gal-positive cells was observed in all the aged somatic cells. F1 intervention in aged cells showed a shift toward fewer positive cells (Figure 4A). The protein levels of p16, p21, and p53 were remarkably enhanced to different levels, as expected, and F1 treatment appeared to show less of an increase in the aged cells (Figure 4B).

To explore how F1 elicits anti-senescence in somatic cells, we tried to identify and profile the 84 genes involved in the cellular senescence pathway with the PCR array method (Figure 4C). A few genes significantly changed in the aged HREC, including upregulated PLAT, CD44, CDKN1A, and SERPINE1 and downregulated SOD1, TERF2, and TERT. In the F1-treated cells, all the changes were less than the denoted threshold (fourfold), indicating effective protection. Compared with the HREC, profound changes were observed in the HCAE and HNA. In the HCAE, during aging, the cellular senescence-associated genes were significantly upregulated in MAP2K6, NOX4, TGFB1I1, EGR1, SERPINB2, IRF5, IRF7, IGFBP5, CDKN1C, MAPK14, COL3A1, MORC3, IGF1, and ING1 and downregulated in CCNA2, CITED2, AKT1, SOD1, CHEK1, TBX2, CDKN2C, PCNA1, and CCND1. These effects were partially or fully restored in the F1-treated cells, with upregulation in TERT and downregulation in RBL1, E2F1, CDKN2D, THBS1, and SPARC. In the HNA, several genes were upregulated in TWIST1, COL3A1, CDKN1A, TGFB1I1, CDKN2B, IGFBP5, THBS1, IGFBP3, and SERPINE1 and downregulated in SOD2, CCNA2, CDKN1C, CCNB1, SOD1, TERF2, IFNG, IGF1, E2F1, TERT, and CDKN2C. Similar to the HCAE pattern, F1 treatment partially or fully repressed the changes. These data suggest that senescence in aged cells involves a reduction in telomere- and cell cycle-associated genes, which are key regulators in cellular senescence and may be the potential targets of F1.

The correlation between the telomere length and shelterin genes was calculated, indicating a strong negative correlation between TRF2 and the telomere length (Figure 4D). Finally, the correlation matrix map of telomere-associated genes and somatic cell type, as well as genes involved in mitochondrial energy metabolism and cellular senescence, was plotted, with a gradient color denoting Spearman’s correlation coefficient (Figure 4E). Those genes in the mitochondrial and senescence pathways were found to be related to each telomere-associated factor manifested by Mantel tests. The different colors denote statistical significance, and the edge width corresponds to Mantel’s r statistic.

### 2.4. TRF2 Depletion-Induced Cellular Senescence

**Figure 5:**
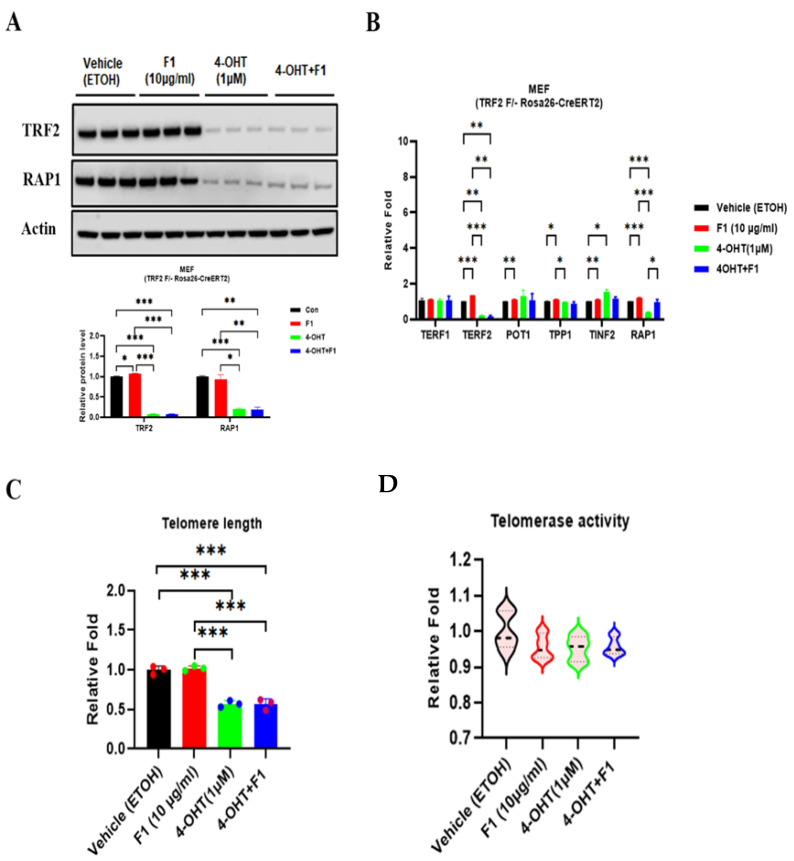
TRF2 is required for F1-mediated telomere protection. (**A**) Immunoblots of TRF2 and RAP1 show efficient depletion in TRF2 by 4-OHT for 7 days in MEF (TRF2 F/- Rosa26-CreERT2) cells. (**B**) qRT-PCR analysis of shelterin proteins. (**C**) Telomere length assay. (**D**) Telomerase activity assay. Results are expressed as mean ± SEM. Statistical significance was determined by one-way ANOVA (* *p* < 0.05, ** *p* < 0.01, and *** *p* < 0.001). We used the Shapiro–Wilk test to test the normality of the distribution. All the data are in accordance with the normal distribution. All the data were compared by one-way analysis of variance followed by a Tukey post hoc test for multiple comparisons.

TRF2 facilitates end protection by sequestrating the terminal telomere repeat sequence within a T-loop structure. This prevents end-to-end fusions in the chromosome and senescence in somatic cells. To investigate the role of TRF2 in cellular senescence, we used an inducible TRF2 ^F/+^ MEF cell line. TRF2 depletion was achieved by 4-hydroxytamoxifen (4-OHT) treatment, which coincided with repressed expression of RAP1 (Figure 5A). Additionally, we further assessed the mRNA levels of other shelterin proteins to confirm any potential effects following TRF2 depletion (Figure 5B). As expected, following 4-OHT induction, we observed reduced TRF2 and RAP1, accompanied by a mild increase. These findings indicate a deficiency in RAP1-TRF2 complex formation. TRF2 depletion did not significantly affect the telomere length or telomerase activity (Figure 5C).

**Figure 6:**
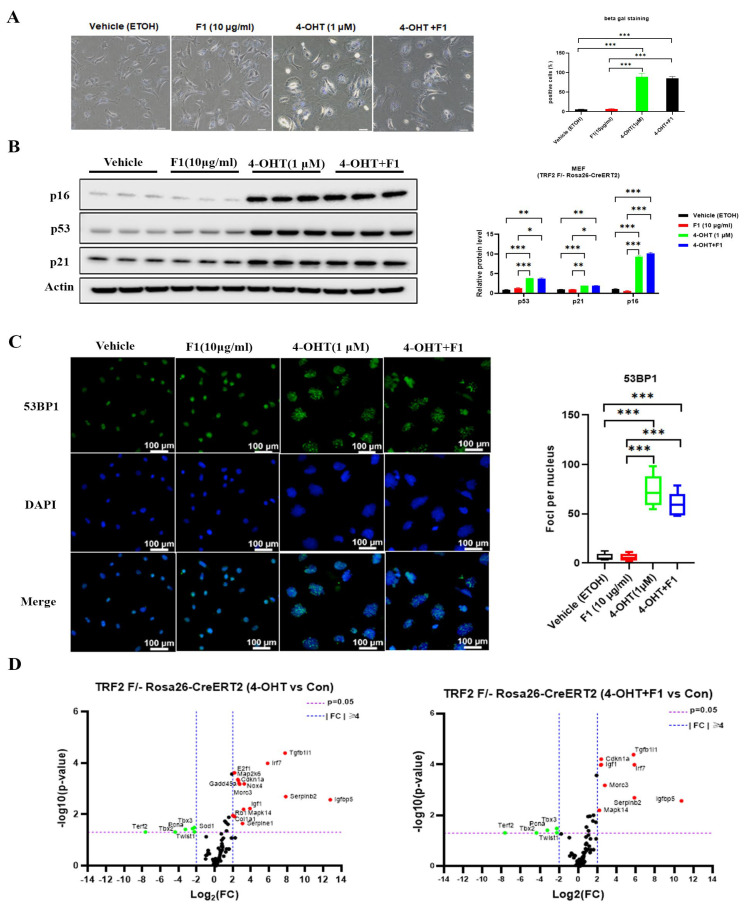
TRF2 is required for F1 to delay cellular senescence. (**A**) Beta gal staining images and quantification. (**B**) Immunoblots of p16 expression. Actin was used as a loading control. (**C**) Representative image of 53BP1 (green) staining of MEF (TRF2 F/- Rosa26-CreERT2) cells after induction with 4-OHT for 7 days. (**D**) PCR array of cellular senescence pathways. Data were normalized with five housekeeping genes. Results are expressed as mean ± SEM. Statistical significance was determined by one-way ANOVA (* *p* < 0.05, ** *p* < 0.01, and *** *p* < 0.001). We used the Shapiro–Wilk test to test the normality of the distribution. All the data are in accordance with the normal distribution. All the data were compared by one-way analysis of variance followed by a Tukey post hoc test for multiple comparisons.

Depletion of TRF2 contributes to a persistent DNA damage response (DDR) during somatic senescence. Unsurprisingly, we observed cellular senescence induction, as evidenced by increased beta galactosidase-positive cells, elevated levels of p53, p21, and p16 protein, and the accumulation of DDR foci marker 53BP1 (Figure 6A–C). To identify and elucidate the underlying mechanism, we performed a PCR array on the senescence pathway. In the TRF2-depleted cells, senescence induction was associated with elevated expression of TGFB1I1, IRF7, E2F1, MAP2K6, CDKN1A, GADD45A, NOX4, MORC3, SERPINB2, IGFBP5, IGF1, RB1, MAPK14, COL1A1, and SERPINE1 and decreased TERF2, TBX2, PCNA, SOD1, TBX3, and TWIST1 expression. However, F1 treatment did not suppress the induction, despite a mild reduction and restoration in certain genes. Considering the genes altered by TRF2 depletion, it is evident that senescence is closely connected to the antioxidant enzyme and cell cycle regulators.

**Figure 7:**
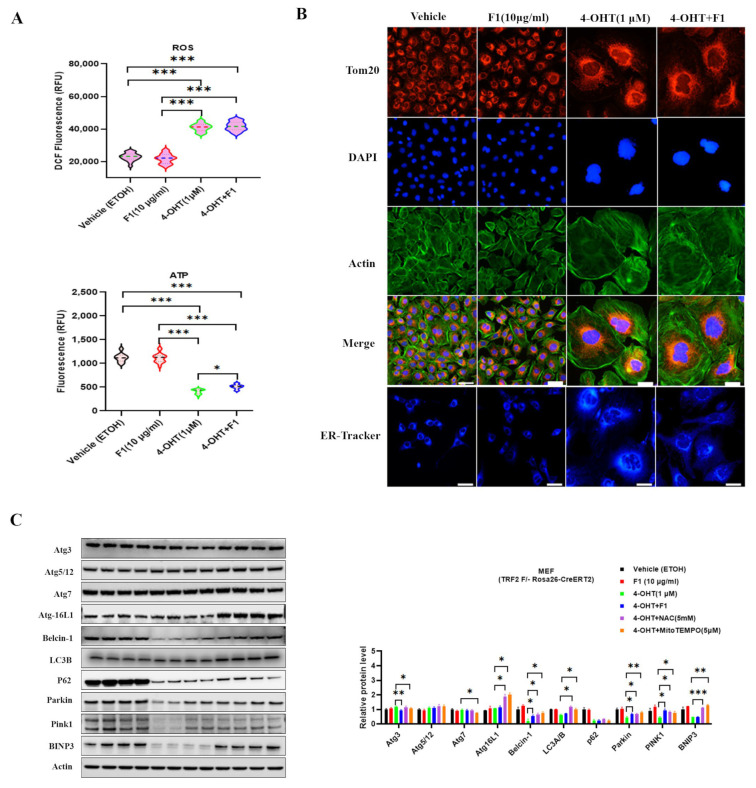
TRF2 is required for F1-mediated mitochondrial protection. (**A**) Qualification of ROS and ATP levels. (**B**) Representative image of Tom20 (red), ER-tracker (blue), and Actin (green) staining of MEF (TRF2 F/- Rosa26-CreERT2) cells after induction with 4-OHT for 7 days. (**C**) Immunoblots for Atg3, Atg5/12, Atg7, Atg16L1, Belcin-1, LC3A/B, p62, Parkin, and PINK1 in extracts from MEF (TRF2 F/- Rosa26-CreERT2) cells pretreated with or without 10 μg/mL F1, 5 mM NAC, and 5 μM MitoTEMPO, followed by induction with 4-OHT for 7 days. Results are expressed as mean ± SEM. Statistical significance was determined by one-way ANOVA (* *p* < 0.05, ** *p* < 0.01, and *** *p* < 0.001). We used the Shapiro–Wilk test to test the normality of the distribution. All the data are in accordance with the normal distribution. All the data were compared by one-way analysis of variance followed by a Tukey post hoc test for multiple comparisons.

We further analyzed the potential effect of TRF2 depletion on mitochondria by evaluating ROS and ATP production. The senescent cells exhibited elevated ROS levels and insufficient ATP production (Figure 7A). F1 treatment appeared to modestly restore ATP production but was much less efficient compared with the normal cells. A substantial proportion of the cells showed an enlarged and flattened morphology, accompanied by accumulated swollen, fragmented mitochondria. Additionally, we observed robust endoplasmic reticulum (ER) expansion in the TRF2-depleted cells, which was not affected by the F1 challenge (Figure 7B). Autophagy is an important cytoprotective program for maintaining cellular health and quality control. To this end, the key factors involved in autophagy were determined by Western blot analysis (Figure 7C). In the TRF2-depleted cells, the levels of Atg3, Atg16L1, and LC3A/B were mildly decreased, while Belcin-1 and p62 were significantly reduced. Due to the accumulation of dysfunctional mitochondria, Parkin and PINK1 were also examined, revealing a notable decrease. Interestingly, this suppression in autophagy (mitophagy excluded) was not prevented by F1 treatment but was effectively restored by NAC (5 mM) and MitoTEMPO (5 µM), which are scavengers of superoxide. These results suggest that the TRF2-depleted cells are involved in the Atg system, Beclin complex, and mitophagy and that ROS plays a driving role for autophagy dysfunction. Together, these data attest to a vital role for TRF2 in safeguarding telomeres against immediate threats, such as DNA damage.

### 2.5. Molecular Docking Simulation in F1 and TRF2

**Figure 8:**
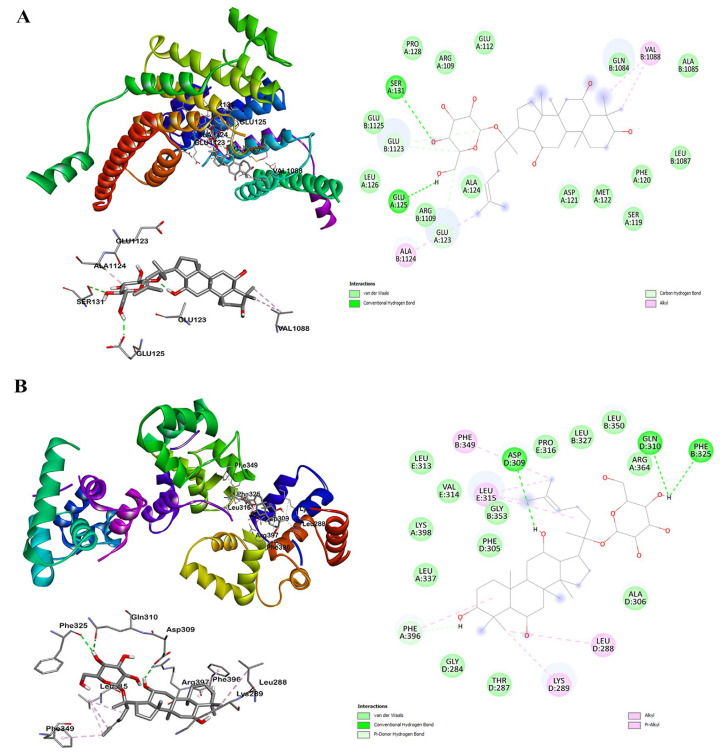
Molecular docking analysis. (**A**) Docking conformation of TRF2 and F1, active site interaction between F1 and amino acid residues of TRF2 dimerization, and interactions on a 2D diagram. (**B**) Docking conformation of TRF2-RAP1 complex and F1, active site interaction between F1 and amino acid residues of TRF2-RAP1 complex, and interactions on a 2D diagram.

We performed molecular docking to identify the potential interactions between TRF2 (PDB code: 1H6P) and the ginsenoside F1 based on the crystal structures (Figure 8A). Docking modeling of F1 into the TRF2 dimerization domain (45E-244L) resulted in a high score of −8.3 kcal/mol. Amino acids involved in the interactions are as follows: ARG109 GLU112 SER119 PHE120 ASP121 MET122 GLU123 ALA124 GLU125 LEU126 THR127 PRO128 SER131 for Chain A and GLN1084 ALA1085 LEU1087 VAL1088 ARG1109 GLU1112 GLU1123 ALA1124 GLU1125 SER1131 for Chain B. We further validated the interaction between TRF2-RAP1 (PDB code: 3K6G) complexes and obtained a score of −9.9 kcal/mol, indicating a good binding force. This interaction was associated with amino acids as follows: LEU337 ARG364 PHE396 ARG397 LYS398 for Chain A, PHE325 LEU327 PHE349 LEU350 ALA351 SER352 GLY353 for Chain B, ILE283 GLY284 THR287 LEU288 LYS289 PHE305 ALA306 ASP309 GLN310 for Chain D, and LEU313 VAL314 LEU315 PRO316 for Chain E. Interestingly, the F1 binding sites were close to the TRF2 dimerization or complex interface without structural interruptions, suggesting a potential enhancement of TRF2 dimerization or complex formation.

## 3. Discussion

The heterogeneous telomere length in somatic cells typically undergoes a decline with advancing age, establishing a natural barrier against tumor growth and contributing to the attrition of aging cells [23]. Our studies uncovered an anti-aging effect of F1, and we propose a conceivable feedback loop whereby compromised telomere induces mitochondrial dysfunction, inducing an increase in ROS generation, decrease in ATP production, and inhibition of mitophagy, culminating in cellular senescence and progressive aging in somatic cells. Additionally, this protective effect is partially favored by a delayed decline in telomerase activities. These findings indicate that beneficial effects of F1 may stem from its improvement in the function of telomeres and mitochondria.

Somatic cells typically exhibit limited proliferative potential, primarily constrained by shortened telomeres [24]. We found significantly compromised telomere in three sequentially grown primary somatic cells characterized by shortened telomere lengths. However, these phenotypes were efficiently rescued by F1. The shelterin complex consists of six proteins: TRF1, TRF2, TIN2, POT1, RAP1, and TPP1. Shelterins bind to telomeric DNA to protect its end and serve as a sensing mechanism for the telomere length [25]. In terms of underlying mechanisms, our investigations elucidated that F1 safeguards telomeres via a reliance on the restoration of shelterin proteins, particularly TRF2, though it is worth noting that the functional relevance of TRF2-mediated telomere protection may exhibit variability across distinct cell types, with pluripotent stem cells showing a dispensable reliance on this mechanism [26]. These findings corroborate previous studies highlighting TRF2 as a key component of telomeric protective factors [27].

Interestingly, our MAPK and AKT phosphorylation antibody arrays revealed a general decline in aged somatic cells when compared with younger counterparts with intact telomeres. These decreases were efficiently inhibited by F1, especially in proteins ERK1/2, MMK6 (S207), MSK2 (S360), CREB (133), HSP27 (S82), AKT (S473), and 4E-BP1 (T36). Although the precise modulation roles of the AKT and MAPK signaling pathways on the telomeres of somatic cells remain to be established, the current studies showed a different phenotypic change occurring in aged somatic cells. These observations align with previous evidence supporting AKT-dependent TRF1 phosphorylation as a mechanism for telomere maintenance. Notably, studies have shown that human cells bearing non-phosphorylatable mutant TRF1 alleles experience accelerated telomere shortening. Similarly, it has been demonstrated that non-phosphorylatable forms of TRF2 trigger telomere uncapping and lead to cellular growth arrest, underscoring the regulatory role of ERK1/2 phosphorylation at serine 323 in telomere stability [28].

As anticipated, our studies revealed conspicuous mitochondrial dysfunction in aged somatic cells: reduced oxidative phosphorylation, decreased ATP production, increased ROS, and diminished sirtuin activity. Our analysis identified the significant downregulation of genes associated with oxidative phosphorylation, including NDUFS6, NDUFS8, NDUFA1, NDUFA8, NDUFA3, NDUFB5, SDHC, COX6C, COX7B, and UQCRC2 in aged HCAE and HREC cells. Furthermore, the mitochondrial genome-encoded genes, namely MitoH2_14573, MitoH2_5726, MitoH2_4162, and itoH2_12106, exhibited noteworthy repression. These pronounced alterations in energy metabolism underlie the observed mitochondrial dysfunction in aged somatic cells, a condition substantially ameliorated by F1 treatment. It is worth noting that previous research has documented that shortened telomeres can suppress the expression of the PGC-1 family of coactivators, potentially resulting in perturbed mitochondrial function [29]. This presents yet another plausible mechanism by which F1 may exert its effects in the context of senescent somatic cells.

The displacement of shelterins and subsequent exposure of the telomere end, which is sensed and recognized by DNA repair machinery as a double-stranded DNA break, result in the telomere shortening. In mammalian cells, the inhibition of shelterin TRF2 induces dysfunctional and uncapped telomeres, triggering a DNA damage response (DDR), which involves 53BP1, phosphorylated histone H2AX, and ATM [30]. The DDR activates transcription factor p53, which is involved in the cell cycle arrest during senescence through regulating p21 in a variety of tissues, contributing to the aging phenotype [31]. It is plausible that under normal conditions, cellular senescence results from telomere shortening, and the loss of TRF2 at telomeres assumes a vital role in aged somatic cells. Accordingly, F1 delays cellular senescence through the maintenance of TRF2 activity. Aside from p53 and p21, other factors involved, such as IGFBP5, IGFBP3, TGFB1I1, SERPINE1, SERPINB2, COL3A1, MAPK14, IRF7, PLAU, and THBS1, were significantly upregulated, while SOD1, SOD2, PCNA, CCNA2, CNB1, and CCND1 were notably downregulated. These changes in gene expression have the potential to directly impair the cell cycle and induce senescence. However, F1 effectively suppressed these alterations to levels comparable to those observed in young somatic cells. Furthermore, we conducted an analysis of the relationship between the telomere factors and three indicators encompassing mitochondrial energy metabolism-related genes, senescence-related genes, and different somatic cell types. Our findings indicate a positive correlation between the levels of telomere markers and genes related to mitochondrial function, while a negative correlation was observed with genes associated with senescence. The significance of the somatic cell type in these correlations was found to be moderate.

TRF2 is the key component of vertebrate telomeres and binds to double-stranded telomeric DNA as a homodimer through mediating homotypic interactions [32]. In our study, we conducted molecular docking simulations and observed that F1 interacts with the TRF2 dimerization domain. The binding of F1 to TRF2 is facilitated by hydrophobic clusters and water-mediated hydrogen bonds. Importantly, these binding sites are in proximity to the TRF2 dimer interface, yet they do not disrupt it. This interaction with F1 may serve to stabilize TRF2 dimerization. However, it is essential to underscore that empirical evidence supporting the binding of F1 to TRF2 and its role in strengthening dimerization warrants further exploration in future research.

**Figure 9:**
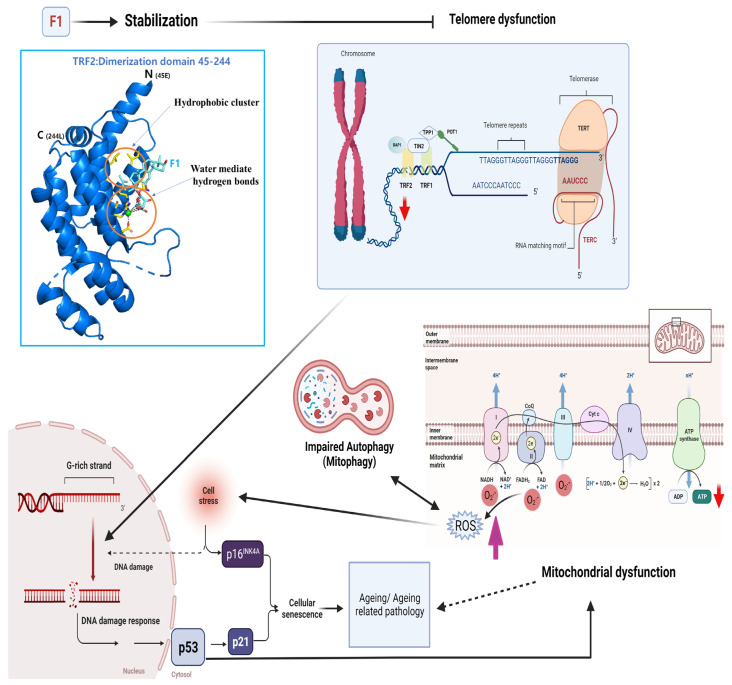
Schematic diagrams of mechanism. Ginsenoside F1 binds TRF2 dimerization close to the interface without any interruption. This binding could potentially stabilize dimerization and delay telomere uncapping. When TRF2 loss starts in the telomere, it may induce a DNA damage response, resulting in p53 activation and leading to cellular senescence through regulation of p21. Activation of p53 can lead to mitochondrial dysfunction, including the production of ROS and a reduction in ATP. Impaired autophagy interacts with damaged mitochondria and indirectly induces cellular senescence.

Mitochondrial dysfunction has been postulated to accelerate telomere shortening and the onset of senescence [33]. Thus, F1 might potentially modulate the maintenance of DDR and senescence occurrence regulated by a feedback loop involving telomere shortening and mitochondrial dysfunction (Figure 9). However, mitochondrial dysfunction could directly cause senescence, and we cannot exclude this action. The reliance on the sole use of three primary somatic cells and the sequential growth induced aging model is one of the limitations herein. Furthermore, F1-associated effects on other telomeric protein complexes such as the heterotrimeric CTC1-STN1-TEN1 (CST) complex, which contributes to telomere attritions, need to be established and delineated. Lastly, the protective effect of F1 on pluripotent human stem cells or in tissues with or without cancer has not been implemented, which is also an important subject for future studies.

In summary, our findings reveal that during the process of normal aging, cells exhibit significant dysfunction in both the telomeres and mitochondria. Importantly, the restoration of TRF2 by F1 not only preserves the functionality of telomeres and mitochondria but also effectively delays cellular senescence.

## 4. Materials and Methods

### 4.1. Reagents

The ethyl alcohol (pure (E7023) and FastStart Essential DNA Green Master (06402712001)) were obtained from Sigma-Aldrich (Darmstadt, Germany). The 16% formaldehyde (methanol-free) was obtained from Thermo Fisher Scientific (28906, Seoul, Republic of Korea). The μ-Dish 35mm image dishes with polymer coverslip bottoms were obtained from ibidi GmbH (Gräfelfing, Germany). N-acetyl-L-cysteine (A7250) and MitoTEMPO (SML0737) were obtained from Sigma-Aldrich (Darmstadt, Germany).

The antibodies against RAP1 were obtained from Bethyl laboratories, Inc. (A300-306A-9, MA, USA); the anti-TPP1 (ab195234), anti-TRF1 (ab10579), anti-p16 (ab211542), and anti-53BP1 (ab175933) were from Abcam (Cambridge, UK); the anti-POT1 (NB500-176) and anti-TRF2 (NB100-56506) were from Novus Biologicals (Littleton, CO, USA); the anti-TIN2 (PA5-67076) was from Invitrogen (Waltham, MA, USA); the anti-p53 (P6874), p16 (ab211542), and anti-actin (A5441) were from Sigma-Aldrich (Darmstadt, Germany); and the anti-P21 (2947), PINK1 (6946), Parkin (4211), autophagy sampler kit (4445), SirT3 (2627), SirT5 (8779), and Tom20 (42406) were obtained from Cell Signaling Technology (Danvers, MA, USA). Ginsenoside F1 was prepared as described in our previous report [34].

### 4.2. Cells and Cell Culture

The normal human astrocyte (CC-2565), cardiac coronary artery endothelial cells (CC-7030), and renal proximal tubule epithelial cells (CC-2553) were obtained from Lonza (Basel, Switzerland). The cells were cultured in systems containing ABMTM Basal Medium (CC-3187) and AGMTM SingleQuotsTM Supplements (CC-4123), EBMTM-2 Basal Medium (CC-3156) and EGMTM-2 MV Microvascular Endothelial Cell Growth Medium SingleQuotsTM supplements (CC-4147), and REBMTM Basal Medium (CC-3191) and REGMTM SingleQuotsTM supplements (CC-4127), respectively. The TRF2 F/- Rosa26-CreERT2 cells were of the mouse embryonic fibroblast cell line (MEF, ATCC, Manassas, VA USA) and were immortalized by SV40-LT antigen and cultured in DMEM (ATCC^®^ 30-2002™) supplemented with 10% fetal bovine serum (FBS; ATCC^®^ 30-2020™), 2 mM L-glutamine (ATCC^®^ 30-2214™), and 0.1 mM nonessential amino acids (NEAA, 100X; Gibco cat 11140050, Seoul, South Korea). TRF2 knockout could be accomplished by Cre recombinase transduction or 1 μM 4-hydroxytamoxifen (4-OHT) treatment for 7 days with the MEFs. All cells were mycoplasma-free before and during the experiments, as determined by a MycoAlert TM Mycoplasma Detection Kit from Lonza.

### 4.3. Cell Aging

All the normal somatic primary cells were serially cultured with or without the ginsenoside F1 (5, 10, and 20 μg/mL) to obtain the required passages (3, 6, 9, and 15). To gather samples for the telomere length and telomerase activity experiments, the cells were divided into two parallel groups. The optimum concentration of ginsenoside F1 was selected and used for the subsequent experiments.

### 4.4. Telomere Length Assay

The genomic DNA of the three somatic cell lines was extracted using a Gentra Puregene Cell Kit (51306, Qiagen, Tegelen, Netherlands) according to the manufacturer’s manual. The relative telomere length was measured using a qPCR assay kit according to the manufacturer’s instructions (8908, M8908, ScienCell Research Laboratories, Inc., Carlsbad, CA, USA), which is designed to directly compare the average telomere lengths of the samples. The reactions were prepared in triplicates containing a genomic DNA template, telomere and single-copy reference primers, FastStart Essential DNA Green Master, and nuclease-free water. Quantitative PCR analyses were performed using a BioradCFX96 Touch Real-Time PCR Detection System. The fold change was calculated by the delta-delta cycle threshold (Ct) method.

### 4.5. PCR Array

The total RNA was extracted from cells with indicated treatments using an RNeasy Mini Kit (74104, Qiagen, Tegelen, Netherlands) and then reverse transcribed to process the cDNA template with an RT^2^ First Strand Kit (330404, Qiagen) following the supplier’s instructions. The mRNA levels of 84 key genes were profiled using an SYBR Green qPCR Mastermix for Human Cellular Senescence RT^2^ Profiler PCR Array (PAHS-050Z, Qiagen) and Human Mitochondrial Energy Metabolism Pathway Plus RT^2^ Profiler PCR Array (PAHS-008YD, Qiagen) according to the manufacturer’s instructions. The array was placed in a Bio-rad CFX96 real-time cycler with the following cycling conditions: 95 °C for 10 min, 95 °C for 15 s, and 60 °C for 1 min with 40 cycles. The data analysis was conducted using a software-based tool that was downloaded from a QIAGEN website. Five housekeeping genes provided within each array were used for normalization.

### 4.6. Antibody Array

The cells were either mock (3) or treated with or without F1 (15) for 3 d and harvested by trypsinization. After removing the remaining PBS, the cells were solubilized with lysis containing a protease and phosphatase inhibitor cocktail. The proteins were collected by centrifugation and determined with the BCA method. A protein concentration of 200 µg/mL for the cell lysates was used to incubate the membrane of the phosphorylation antibody array (AAH-PPP, RayBiotech, Norcross, GA, USA) according to the manufacturer’s protocols. The membranes were incubated with an antibody cocktail followed by HRP-anti-Rabbit IgG. Signals were detected with a chemiluminescence imaging system.

### 4.7. Western Blot

The cell extracts were isolated with a Minute™ Total Protein Extraction Kit (SD-001/SN-002, Invent Biotechnologies, Inc., Waltham, MN, USA) according to the provider’s manual. The extracts (20 μg) were separated on Bolt^TM^ 10%, Bis-Tris Plus, mini protein gel (NW00105BOX, Thermo Fisher Scientific) and then transferred to PVDF membranes using an iBlot2 Transfer stack and device (IB24002 and B21001, Thermo Fisher Scientific). The membranes were blocked for 1 h at room temperature (P/N: 927-60001) and incubated overnight at 4 °C on a rocker with primary antibodies in the buffer (P/N: 927-65001) provided by LI-COR (NE, USA). After washing with TBST three times for 10 min each, the membranes were incubated with HRP-tagged secondary antibodies (7074 and 7076) for 2 h at room temperature. Next, the membranes were developed with chemiluminescence using Amersham ECL detection reagent (RPN3004, Cytiva, Marlborough, MA, USA) with an Odyssey Fc Imager (LI-COR).

### 4.8. Intracellular Superoxide Detection

The cells were cultured at a low density and quiesced for 1 day with a low-serum medium. The general oxidative stress was measured using CM-H_2_DCFDA. For the assay, 5 μM CM-H_2_DCFDA diluted in Hanks’ balanced salt solution (HBSS) buffer was added onto the cells and incubated for 30 min. The cells were then washed and quantitated by the measurement of the fluorescence density in a microplate reader (Spark, Tecan, Männedorf, Switzerland) with an excitation of 492 nm and emission of 517 nm. Data were expressed in relative fluorescence units (RFUs) and presented as mean ± SEM.

### 4.9. ATP Assay

The levels of ATP in the live cells were determined with a luminescent assay kit (ab113849, Abcam). Briefly, cells seeded in 96-well microplates were grown in 100 μL of media per well before the assay. Then, 50 μL of detergent was added into each well, and we sealed and shook the plates for 5 min at 600–700 rpm. After that, we added 50 μL substrate solution to each well and repeated the shaking. We adapted the plate to darkness by covering it for 10 min and measured the luminescence immediately with a microplate reader.

### 4.10. Senescence-Associated β-Galactosidase (SA-βG) Staining

The cells with the indicated treatments were washed with PBS, fixed for 10–15 min at room temperature, and rinsed twice with PBS. Next, β-galactosidase staining solution was added and incubated at 37 °C at least overnight in a dry incubator. Positive cells with a developed blue color were observed under a microscope (200× total magnification) and counted (ECLIPSE Ti2-U, Nikon, Tokyo, Japan).

### 4.11. Real-Time qPCR

The total RNA was extracted from the cells with an RNeasy Kit (Qiagen) and reverse transcribed using the cDNA template with a RT^2^ First Strand Kit (Qiagen) according to the manufacturer’s protocols. Quantitative real-time PCR was performed with a CFX96 Touch system (Bio-Rad Laboratories, Hercules, CA, USA) using SYBR Green qPCR Mastermix (Qiagen) according to the manufacturer’s instructions. The samples were run in triplicate. The primers are listed in the supplements.

### 4.12. Immunofluorescence

The cells with indicated treatments and at 60% confluence in the imaging dishes were fixed in 4% paraformaldehyde for 15 min and washed with PBS three times. The cells were then permeabilized with pre-chilled 80% ethanol at −20 °C for 30 min, rinsed in cold acetone, air dried, and blocked with a 5% BSA/PBS solution for 1 h. The cells were incubated with the indicated primaries overnight at 4 °C, followed by secondary Alexa-488/594-conjugated antibodies (Invitrogen, Waltham, MA, USA) for 1 h. After three washes with PBS, the cells were then incubated with DAPI solution (Invitrogen) for 5 min. Images were captured with a fluorescence microscope (Nikon). Positive stainings were counted in three independent experiments. At least 80 cell nuclei in each group were selected for statistical measurements.

### 4.13. Molecular Docking

The structures of the TRF2 (PDB code: 1H6P) and TRF2-RAP1 (PDB code: 3K6G) complexes were downloaded from the protein databank (PDB) (https://www.rcsb.org; accessed on 15 July 2023). The structure of ginsenoside F1 was from the National Center for Biotechnology Information (NCBI) PubChem compound database (http://www.ncbic.nlm.nih.Gov/pccompound; accessed on 15 July 2023). F1 was docked onto the binding pockets of the TRF2 and TRF2-RAP1 complexes using SwissDock (http://swissdock.ch/; accessed on 15 July 2023) integrated with UCSF Chimera Alpha v1.16 (RBVI, San Francisco, CA, USA). The binding affinity between targets was expressed as the lowest energy score (kcal/mol) in the simulation.

### 4.14. Statistical Analysis

The correlations were examined using R software (https://www.r-project.org/; accessed on 15 July 2023) and visually analyzed as demonstrated in previous reports [35]. The data were analyzed using one-way ANOVA for repeated experiments, followed by Tukey’s post hoc pairwise multiple comparisons (homogeneous variance) using Prism 9 (GraphPad). A *p* value < 0.05 was considered significant. The mean ± SEM is plotted in all the bar graphs. All the experiments were performed at least three times.

## Data Availability

All data are available within the article. Other data are available from the corresponding author upon reasonable request.
